# A mutant complement factor H (W1183R) enhances proteolytic cleavage of von Willebrand factor by ADAMTS-13 under shear

**DOI:** 10.1016/j.jtha.2024.11.031

**Published:** 2025-01-09

**Authors:** Wenjing Cao, Yi Liu, X. Frank Zhang, X. Long Zheng

**Affiliations:** 1 Department of Pathology and Laboratory Medicine, The University of Kansas Medical Center, Kansas City, KS 66160, USA; 2 Institute of Reproductive Medicine and Developmental Sciences, The University of Kansas Medical Center, Kansas City, KS 66160, USA; 3 Department of Biomedical Engineering, University of Massachusetts Amherst, Amherst, MA 01003, USA

**Keywords:** ADAMTS-13, complement factor H, mechanical force, protein-protein interaction, von Willebrand factor

## Abstract

**Background:**

A loss-of-functional mutation (W1183R) in human complement factor H (CFH) is associated with complement-associated hemolytic uremic syndrome; mice carrying a similar mutation (W1206R) in CFH also develop thrombotic microangiopathy but its plasma von Willebrand factor (VWF) multimer sizes were dramatically reduced. The mechanism underlying such a dramatic change in plasma VWF multimer distribution in these mice is not fully understood.

**Objectives:**

To determine the VWF and CFH interaction and how CFH proteins affect VWF multimer distribution.

**Methods:**

We employed recombinant protein expression, purification, and various biochemical and biophysical tools.

**Results:**

Purified recombinant W1183R-CFH but not wild-type (WT) CFH protein enhanced the proteolytic cleavage of both peptidyl and multimeric VWF substrates by recombinant ADAMTS-13 in a concentration-dependent manner. Microscale thermophoresis assay demonstrated that both W1183R-CFH and WT-CFH proteins bound various VWF fragments (eg, AIM-A1, A1-A2-A3, D’D3, D’D3-A1, and D’D3-A1-A2) with high affinities. Optical tweezer experiments further showed a concentration-dependent alteration in the contour length (L_c_) and the persistent length (L_p_) following pulling VWF-A2 domain in the presence of W1183R-CFH or WT-CFH protein. AlphaFold experiments revealed conformational changes in the VWF-A2, particularly the central region where the cleavage bond resides following addition of W1183R-CFH or WT-CFH protein.

**Conclusion:**

These results demonstrate for the first time that W1183R-CFH but not WT-CFH protein enhances the proteolytic cleavage of VWF by ADAMTS-13 under shear. This may be achieved by mechanic-induced conformational changes of the central A2 domain, leading to an enhanced cleavage of Tyr^1605^-Met^1606^ bond by ADAMTS-13 under pathophysiological conditions.

## 1 | INTRODUCTION

Complement factor H (CFH), a 150-kDa plasma protein, is primarily synthesized in hepatocytes [1] and endothelial cells [2]. It is a key negative regulator of complement activation in the alternative pathway [3]. CFH serves as a cofactor for complement factor I (protease) to inactivate C3b, thus dampening the complement activation [4]. Loss-of-functional mutations of *CFH* in humans [3,5] or in mice [6,7] result in complement-associated hemolytic uremic syndrome (cHUS), also known as atypical hemolytic uremic syndrome (aHUS) [8]. Patients with cHUS develop thrombocytopenia, microangiopathic hemolytic anemia, and organ damage [9]. Renal failure or end-stage renal disease may develop, or death may occur if the disease is not recognized and managed promptly [3,10,11]. Complement inhibition with an anti-C5 monoclonal antibody such as eculizumab has been shown to be highly efficacious for managing cHUS and reducing mortality [12–14].

ADAMTS-13, a 195-kDa plasma protease [15–17], is synthesized in hepatic stellate cells [18,19] and endothelial cells [20–22]. Its main function is to cleave endothelium-anchored ultralarge (UL) von Willebrand factor (VWF) [23,24] and circulating large VWF multimers under flow [25,26]. This proteolytic cleavage reduces the adhesiveness of VWF multimers, thus preventing excessive VWF–platelet interaction and the formation of thrombosis in small arterioles and capillaries. Severe deficiency of plasma ADAMTS-13 activity, resulting from either hereditary mutation on *ADAMTS-13* or acquired autoantibodies, leads to the development of disseminated platelet- and VWF-rich microvascular thrombosis, the characteristic feature of thrombotic thrombocytopenic purpura (TTP) [27,28]. Compared with cHUS, patients with TTP usually present less severe renal dysfunction (eg, serum creatinine usually <2.0–2.2 mg/dL) but more severe thrombocytopenia (eg, platelet count often <30 × 10^9^/L) [27]. Most importantly, plasma ADAMTS-13 activity is severely deficient (ie, <10 U/dL or <10% of normal) in TTP [29–31] but relatively normal in cHUS [30]. So, plasma ADAMTS-13 assessment is the key for differential diagnosis of thrombotic microangiopathy.

We have previously found that mice carrying a heterozygous mutation in complement factor H (*cfh*) (eg, changing amino acid residue from Trp (W) to Arg (R) at the position of 1206, corresponding to the position of 1183 in humans) did not develop cHUS unless severe deficiency of plasma ADAMTS-13 activity is also present [7]. Mice with such a homozygous *cfh* mutation (*cfh*^*R/R*^*)* or a heterozygous *cfh* mutation (*cfh*^*W/R*^) plus *Adamts-13*^*−/−*^ did develop thrombocytopenia, an increased level of lactate dehydrogenase and creatinine, and occlusive thrombi in small arterioles and capillaries, consistent with cHUS [6,7]. Intriguingly, the mice carrying the homozygous *cfh* mutation (*cfh*^*R/R*^) in the presence of normal Adamts-13 activity exhibited a dramatically reduced size of plasma VWF multimers despite an increased level of plasma VWF antigen [6,7] when compared with those in *WT* and heterozygous *cfh mutant* (*cfh*^*W/R*^) mice. Such a dramatic reduction of plasma VWF multimer size was not observed in the *Adamts-13*^*−/−*^ mice [7], suggesting that Adamts-13 is required for proteolysis of VWF. However, the molecular mechanism underlying such a homozygous mutation in *cfh* that affects plasma VWF multimer distribution is not fully understood.

Previous studies have shown that CFH interacts with VWF, but the data are conflicting, largely due to the difference in the CFH preparations. One study showed that plasma-derived CFH enhances the cleavage of VWF by ADAMTS-13 [32] but another study reported an opposite effect, suggesting that CFH inhibits the proteolytic cleavage of VWF by ADAMTS-13 [2]. However, all these assays were performed under a nonphysiological condition (eg, no shear and prolong incubation). In addition, plasma-derived CFH is shown to have a disulfide bond-reducing activity, which is independent of ADAMTS-13 activity. However, such an activity is only observed after plasma CFH is heat-treated or incubated with EDTA [33]. These conflicting results, plus surprising findings of significant reduction of plasma VWF multimer size in the homozygous (*cfh*^*R/R*^) mice, prompted us to further investigate the interactions between CFH and VWF, and how CFH binding to VWF affects its proteolysis by ADAMTS-13 under shear, and what the molecular mechanism underlying such an effect could be. We believe that the results may shed new light on how CFH and its pathologic mutants may affect VWF homeostasis and thrombosis under various pathophysiological conditions.

## 2 | METHODS

### 2.1 | Constructs and stable cell lines

The *pCMV3-WT-CFH-V5-His* was constructed by inserting a human full-length *CFH* cDNA into the *pCMV3* vector (Sino Biological). The *pCMV3-W1183R-CFH-V5-His* was constructed by inserting a synthetic DNA fragment containing the W1183R mutation (changing TGG to CGG) in the *CFH* gene into a parental pCMV3-WT-CFH-V5-His following the cleavage by PshA I and XhoI. Both constructs contain a neomycin-resistant gene, a 15-nucleotide linker (GGGGGTGGAGGCTCT), and a V5-His epitope at the C-terminus. The constructs were sequenced to confirm the accuracy.

Human embryonic kidney cells (HEK-293) were cultured with a Dulbecco-modified eagle medium (Invitrogen) containing 10% of fetal bovine serum (ThermoFisher). The cells were transfected with a mixture of PolyJet and plasmid DNA at a ratio of 3:1 (vol/wt) in a serum-free Dulbecco-modified eagle medium with high glucose. After 48 hours of transfection, stable clones were selected following treatment of transfected cells with geneticin (G418) (0.5 mg/mL) (Invitrogen). Those with high expression of WT-CFH or W1183R-CFH were identified by Western blotting using a polyclonal anti-CFH IgG (Invitrogen) as described previously [34,35].

### 2.2 | Expression of recombinant VWF fragments

Human VWF fragments containing a short A1 fragment (A1-S) (aa1261–1468) and A1-A2-A3 fragment were purchased from ImmunoPrecise. Human VWF-A1 fragment containing an autoinhibitory motif (AIM-A1) (aa1238–1481) and human VWF-A2 fragment were expressed in HEK293 cells and purified to homogeneity as previously described [36]. For laser tweezer experiments, the human VWF-A2 fragment was engineered to contain an Avi-His tag at its N-terminus and a SpyTag at its C-terminus, as described elsewhere [37,38]. Human VWF D’D3, D’D3A1, and D’D’A1A2 fragments were expressed in *Escherichia coli,* purified by Ni-affinity chromatography, and refolded as described previously [36]. The purified VWF-A2 fragment was further biotinylated. The carboxyl-polystyrene sphere beads (2 μm in diameter), from Spherotech, were covalently coupled with streptavidin by a PolyLink coupling kit as previously described [39].

### 2.3 | Preparation of recombinant CFH and ADAMTS-13 proteins

Both human recombinant WT-CFH and W1183R-CFH were expressed in HEK293 cells [35,40] and purified by an ion exchange (Q-fast) followed by a Ni-NTA affinity column (GE health). The concentration of the purified CFH proteins was determined by absorbance of 280 nm (corrected with light scattering at 340 nm) with extinction coefficients of 0.97 [41]. Recombinant ADAMTS-13 was also expressed in HEK293 cells and purified similarly as previously described [35,42]. Sodium dodecyl sulfate-polyacrylamide gel under denaturing and reducing conditions followed by Coomassie blue staining determined the purity and integrity of purified recombinant CFH proteins.

### 2.4 | Proteolytic cleavage of the fluorescence-energy resonance transfer VWF73 substrate

The proteolytic cleavage of a fluorescence-energy resonance transfer (FRETS)-VWF73 by ADAMTS-13 in the absence or presence of a CFH protein was performed according to the protocol described previously [43,44]. Briefly, a recombinant ADAMTS-13 (12.5 nM) was mixed with WT-CFH or W1183R-CFH (0–500 nM). A FRETS-VWF73 substrate (final, 2 μM) in 5 mmol/L Bis-Tris, pH 6.0, 25 mmol/L CaCl_2_, and 0.005% Tween-20 at 25 °C was added. The rate of fluorescence generation per second was monitored at a 2-minute interval for 60 minutes on a Gemini XPS microtiter plate reader (Molecular Devices) using excitation 485 nm and emission 530 nm at 25°C. The relative activity (%) was determined based on the standard curve generated with a known concentration of recombinant ADAMTS-13 protease.

### 2.5 | Proteolytic cleavage of a multimeric VWF under shear

Purified human recombinant VWF (37.5 μg/mL) was mixed with recombinant ADAMTS-13 (50–100 nM) in the absence or presence of an increasing concentration of WT-CFH or W1183R-CFH protein (0–2 μM) in 20 mM HEPES buffer containing 1 mg/mL bovine serum albumin, 150 mM NaCl in a 0.2 mL thin-walled polymerase chain reaction tube (Fisher Scientific). The reaction mixture (total volume, 20 μL) was incubated at 25 °C for 5 minutes and then subjected to vortexing rotation at 2500 rpm for 5 minutes using a bench-top mini vortexer (Fisher Scientific) as previously described [40,45]. The reaction was quenched by an addition of sample buffer (Bio-Rad) without β-mercaptoethanol and denatured at 95 °C for 10 minutes. The proteins were fractionated on 5% Sodium dodecyl sulfate-polyacrylamide gel at 120V for 150 minutes and transferred onto a nitrocellulose membrane. VWF and its cleavage products on the membrane were detected by an incubation with a rabbit anti-VWF IgG (Agilent Dako) diluted with Tris-buffered saline containing 1% casein (1:5000), followed by an incubation with an infrared fluorescein-labeled anti-rabbit IgG (1:10 000) (Li-COR Biosciences). The fluorescent signal was obtained by an Odyssey imaging system and converted into gray image as previously described [45,46].

### 2.6 | CFH and VWF binding kinetics by monolith microscale thermophoresis

The physical binding kinetics between VWF and CFH proteins were assessed by microscale thermophoresis (MST), which detects the motion of fluorescent molecules along a microscopic temperature gradient applied to the glass capillary filled with different concentrations of target molecules, which reflects changes in the molecular hydration shell, charge, or size upon the occurrence of binding interactions between molecules. Briefly, human recombinant VWF fragments including A1-S, AIM-A1, A2, A1A2A3, D’D3, D’D3A1, and D’D3A1A2 were fluorescein-labeled with the monolith protein labeling kit (RED-NHS 2nd generation, MO-L011). The WT-CFH or W1183R-CFH was prepared by a 15-step 2-fold dilution in which the concentration in the last step was reduced to 1/215 of its original concentration. After mixing the fluorescein-labeled VWF fragments with a serially diluted CFH sample, the mixed samples were loaded into the monolith premium capillaries (MO-K025) (FisherScientific). The changes in the MST signal upon the binding of a ligand and a target are quantified. The dissociation constant (*K*_*d*_) was obtained by fitting a dose-response curve to a saturation curve. The Hill model was used for affinity quantification of multivalent interactions in the experiments [47].

### 2.7 | Optical tweezer pulling assay

To determine how CFH binding to VWF-A2 affects the A2 conformation, we performed the optical laser tweezer experiments. Briefly, the streptavidin-coated carboxyl-polystyrene beads were incubated with a biotinylated VWF A2 fragment and a SpyCatcher-coupled DNA handle separately before testing [36]. After injection of 2 different premixed beads into the reaction chamber, one was aspirated and fixed by a micropipette, while the other bead was trapped and controlled by dual laser traps. The bead-protein-DNA handle-bead connection system would be formed between 2 beads when approaching one to the other. Different concentrations of WT-CFH or W1183R-CFH were added to the reaction chamber separately. The most probable unfolding extensions were measured at 5 different speeds. The pulling experiments of A2 alone, A2 unfolding with presence of WT-FH, and A2 unfolding with W1183R-CFH collect a total of 330, 383, and 499 individual pulling traces, respectively. The applied forces and positions of the laser-trapped bead were recorded while altering the distance between the 2 beads. The persistent length and contour length were obtained by fitting the worm-like chain (WLC) model [48–50] to the force-extension data, while the unbinding rate and barrier position can be solved by fitting the Bell-Evens model [51,52] to the force and loading rate data.

## 3 | RESULTS

### 3.1 | Expression, purification, and characterization of recombinant CFH proteins

**FIGURE 1:**
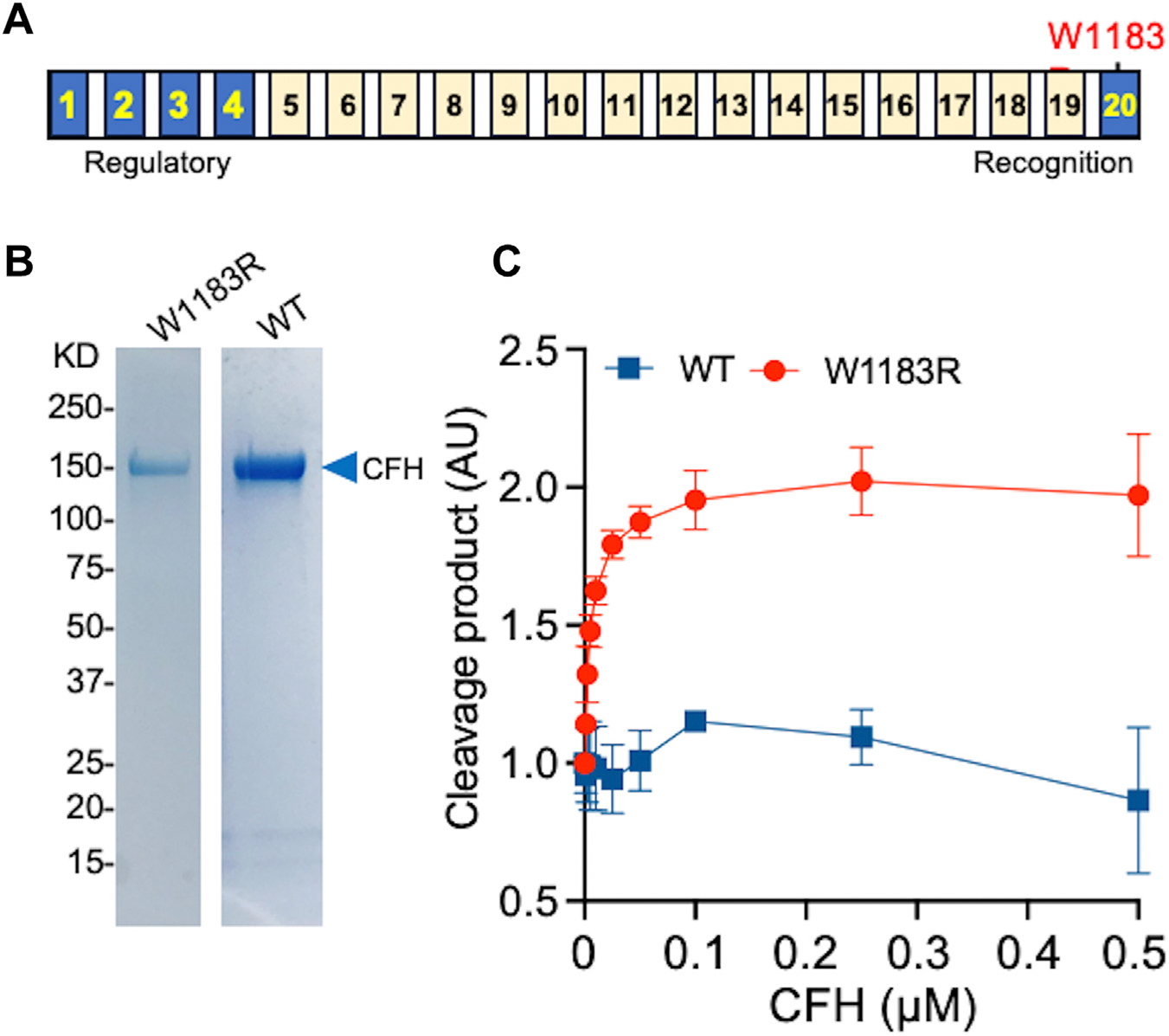
Preparation and characterization of recombinant complement factor H (CFH). (A) Schematic domain structure and location of the mutation of recombinant human CFH. (B) Sodium dodecyl sulfate-polyacrylamide gel with Coomassie blue staining detects the purified mutant (W1183R) and wild-type CFH (150-kDa, arrowhead). (C) Effect of recombinant wild-type and mutant (W1183R) CFH on the proteolytic cleavage of FRETS-VWF73 by recombinant ADAMTS-13. The data shown are the mean and standard deviation of the mean (SD) from 4 independent experiments (*n* = 4).

Human recombinant WT-CFH and W1183R-CFH proteins (Figure 1A) tagged with a V5-His were expressed in stable transfected HEK293 cells and purified to homogeneity by Q-fast and Ni-affinity chromatograph. Both WT-CFH and W1183R-CFH proteins assessed by Sodium dodecyl sulfate-polyacrylamide gel electrophoresis with Coomassie blue staining migrated at the 150-kDa marker (Figure 1B), consistent with the molecular weight of the full-length CFH proteins.

### 3.2 | W1183R-CFH but not WT-CFH enhanced the cleavage of FRETS-VWF73 by ADAMTS-13

To determine if CFH affects proteolytic cleavage of VWF, we first performed the FRETS-VWF73 assay using a peptidyl substrate containing the cleavage bond (Tyr-Met) derived from the central A2 domain. As shown, the rate of cleavage of the FRETS-VWF73 by ADAMTS-13 increased as a function of concentration of mutant W1183R-CFH but not WT-CFH protein. The maximal fold of increase was about 2 for W1183R-CFH, but no significant difference in ADAMTS-13 cleaving activity in the presence of various concentrations of WT-CFH up to 0.5 μM (*P* = .66) (Figure 1C). Either a W1183R-CFH or WT-CFH in the absence of recombinant ADAMTS-13 did not result in proteolytic cleavage of FRETS-VWF73 (not shown), suggesting no ADAMTS-13 contamination in our purified recombinant CFH proteins. These results demonstrate that W1183R-CHF, but not WT-CFH protein, enhances the proteolytic cleavage of a peptidyl VWF substrate by ADAMTS-13.

### 3.3 | W1183R-CFH but not WT-CFH enhances the proteolytic cleavage of multimeric VWF by ADAMTS-13 under shear

**FIGURE 2:**
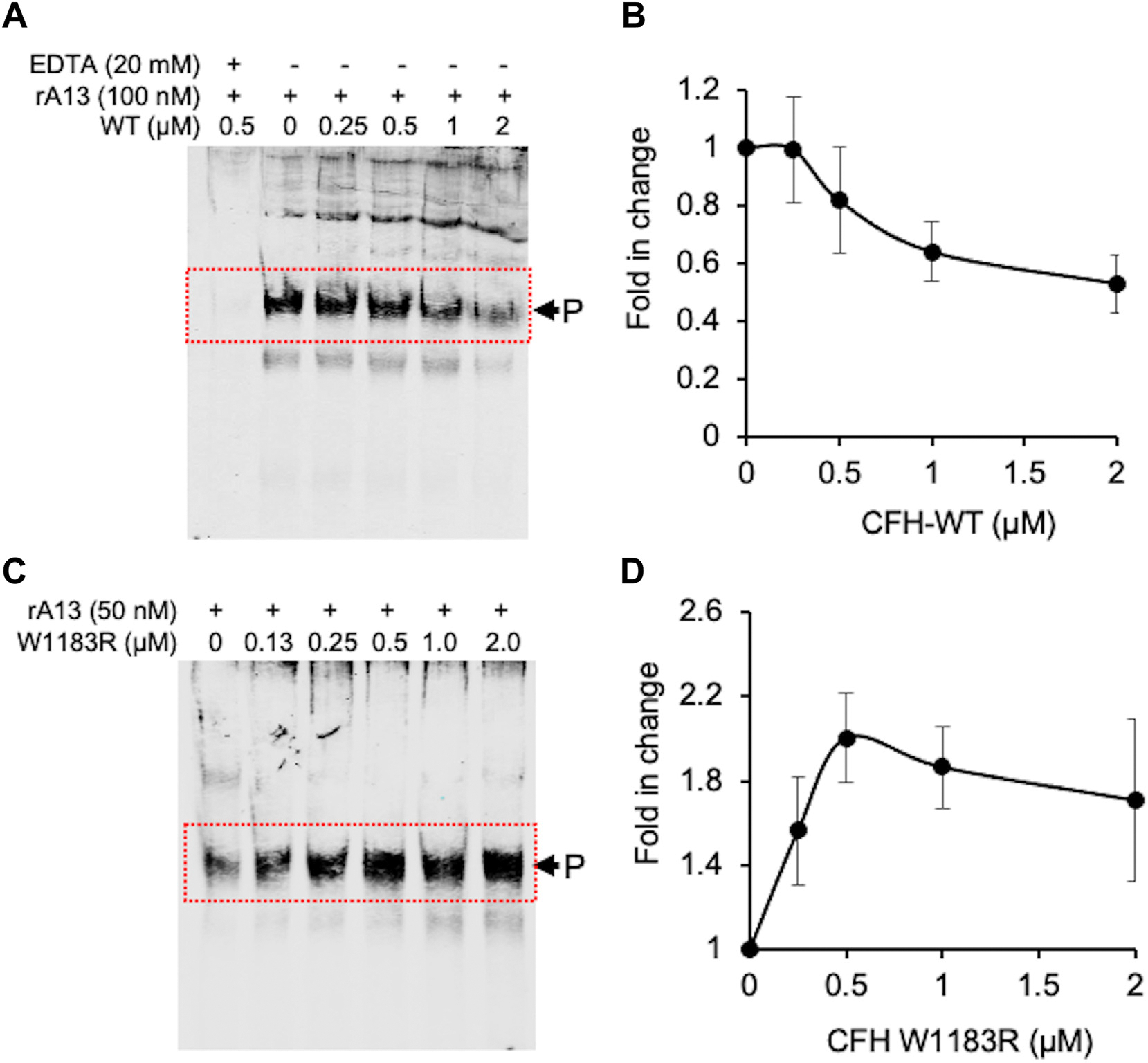
Effect of recombinant wild-type and mutant complement factor H (CFH) on proteolytic cleavage of multimeric von Willebrand factor (VWF) by ADAMTS-13 under shear. (A, B) Western blotting and quantitation, respectively, of the cleavage product (~350-kDa, arrowhead) of recombinant multimeric VWF by recombinant ADAMTS-13 (rA13) under vortexing-induced shear (2500 rpm) in the absence or presence of various concentrations of wild-type CFH. (C, D) Western blotting and quantitation, respectively, of the cleavage product as a function of concentrations of W1183R-CFH. The data shown are the mean and standard deviation of the mean (SD) from 4 independent experiments (*n* = 4). CFH, complement factor H; VWF, von Willebrand factor.

To determine if recombinant CFH proteins modulate the proteolytic cleavage of multimeric VWF by ADAMTS-13 under more physiological conditions, we employed the vortexing-based assay in which an arterial shear was introduced following a vortexing rotation at 2000 rpm [53]. As shown, in the presence of an increasing concentration of WT-CFH protein, the intensity of the proteolytic cleavage product (~350 kD band) was modestly reduced in a concentration-dependent manner (Figure 2A, B). In contrast, in the presence of an increasing concentration of the W1183R-CFH protein, the proteolytic cleavage product was significantly increased as a function of an increasing concentration of the W1183R-CFH protein with the maximal enhancement of ~2 fold (Figure 2C, D). No cleavage product was observed when EDTA was present, which blocks ADAMTS-13 activity (Figure 2A), suggesting that CFH protein itself has no proteolytic or reductase activity under the assay conditions. Our results further support the hypothesis that WT-CFH protein may inhibit VWF proteolysis by ADAMTS-13, but W1183R-CFH protein enhances it under physiological shear.

### 3.4 | CFH proteins bound various fragments of VWF with high affinities

**FIGURE 3:**
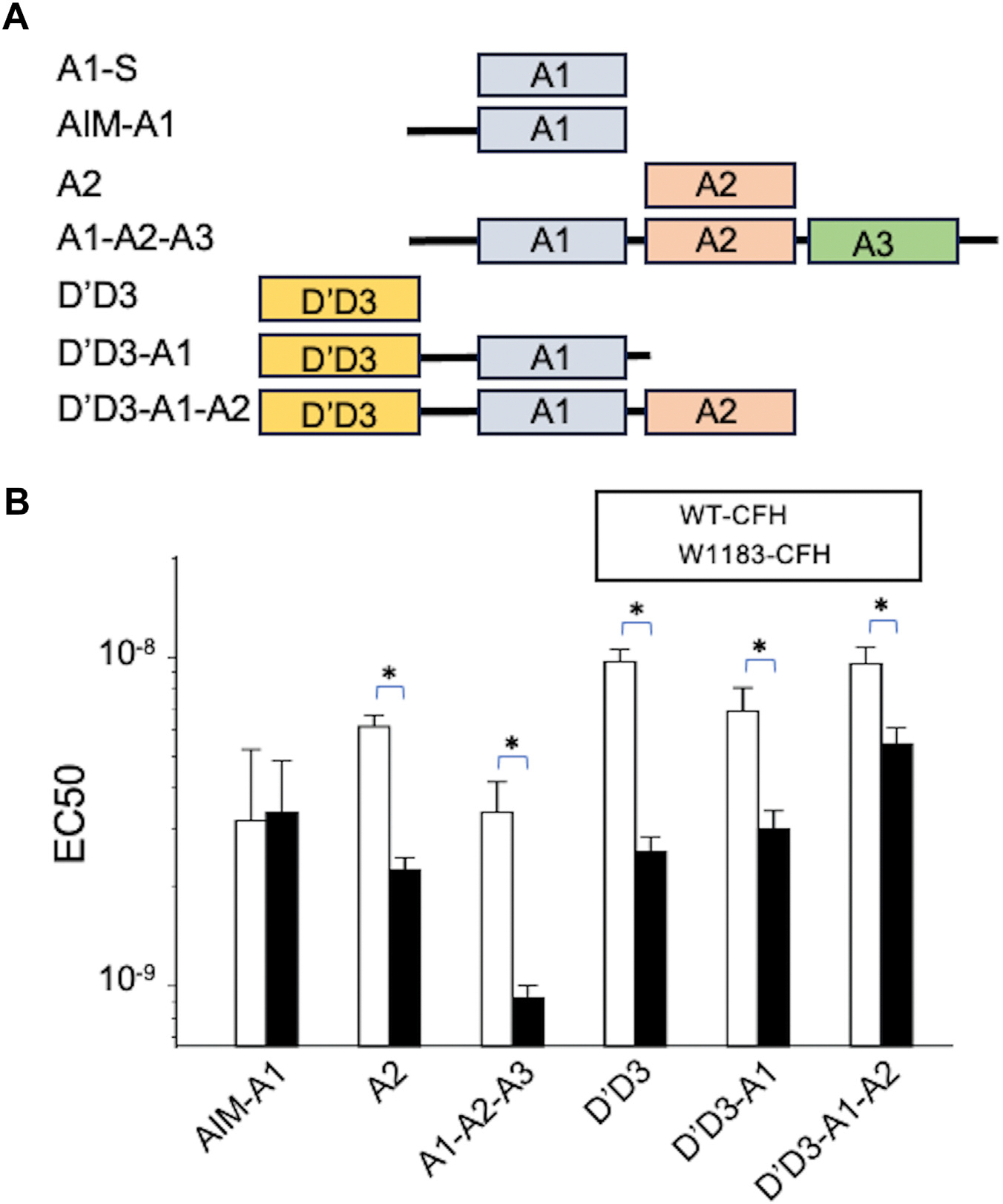
Binding of various von Willebrand factor (VWF) fragments to complement factor H (CFH) protein by microscale thermophoresis. (A) Schematic domain structure of various VWF fragments including A1-S (lacking the N-terminal autoinhibitory module, AIM), AIM-A1 (containing AIM), A2, A1-A2-A3, D’D3, D’D3-A1, and D’D3-A1-A2. (B) Binding affinities (half maximal effective concentrations [EC_50_]) between wild-type CFH (white bars) or mutant CFH (solid bars) and various VWF fragments as illustrated in the panel A. Data shown are the mean ± standard deviation of the mean (SD) from 3 independent experiments (*n* = 3). Mann–Whitney U-test was performed to determine the statistical significance. Here, * indicates *P* < .05. CFH, complement factor H; VWF, von Willebrand factor.

To determine the binding sites and affinities of CFH proteins to VWF, we performed the MST assay [47]. The gradual changes of MST signal resulting from a titration of a ligand were fitted into the Hills model, which yielded the half maximal effective concentrations (EC_50_) of CFH proteins for various VWF fragments. The results demonstrated that both WT-CFH and W1183R-CFH proteins bound to VWF fragments including AIM-A1, A2, A1-A2-A3, D’D3, D’D3-A1, and D’D3-A1-A2 with high affinities [sub-nanomolar EC_50_(s)] (Figure 3A,B and Table 1). The W1183R-CFH bound to most VWF fragments except for AIM-A1 with higher affinities than WT-CFH did. No binding was observed between CFH proteins (either WT or W1183R) and A1-S that lacks the AIM segment (D1261-T1468) (Figure 3B and Table 1). These results suggest that the VWF D’D3, AIM, and A2, but not A1, contain the high-affinity CFH binding sites.

### 3.5 | Optical tweezer analysis demonstrated the potential force-induced conformational changes of A2 triggered by CFH proteins

**FIGURE 4:**
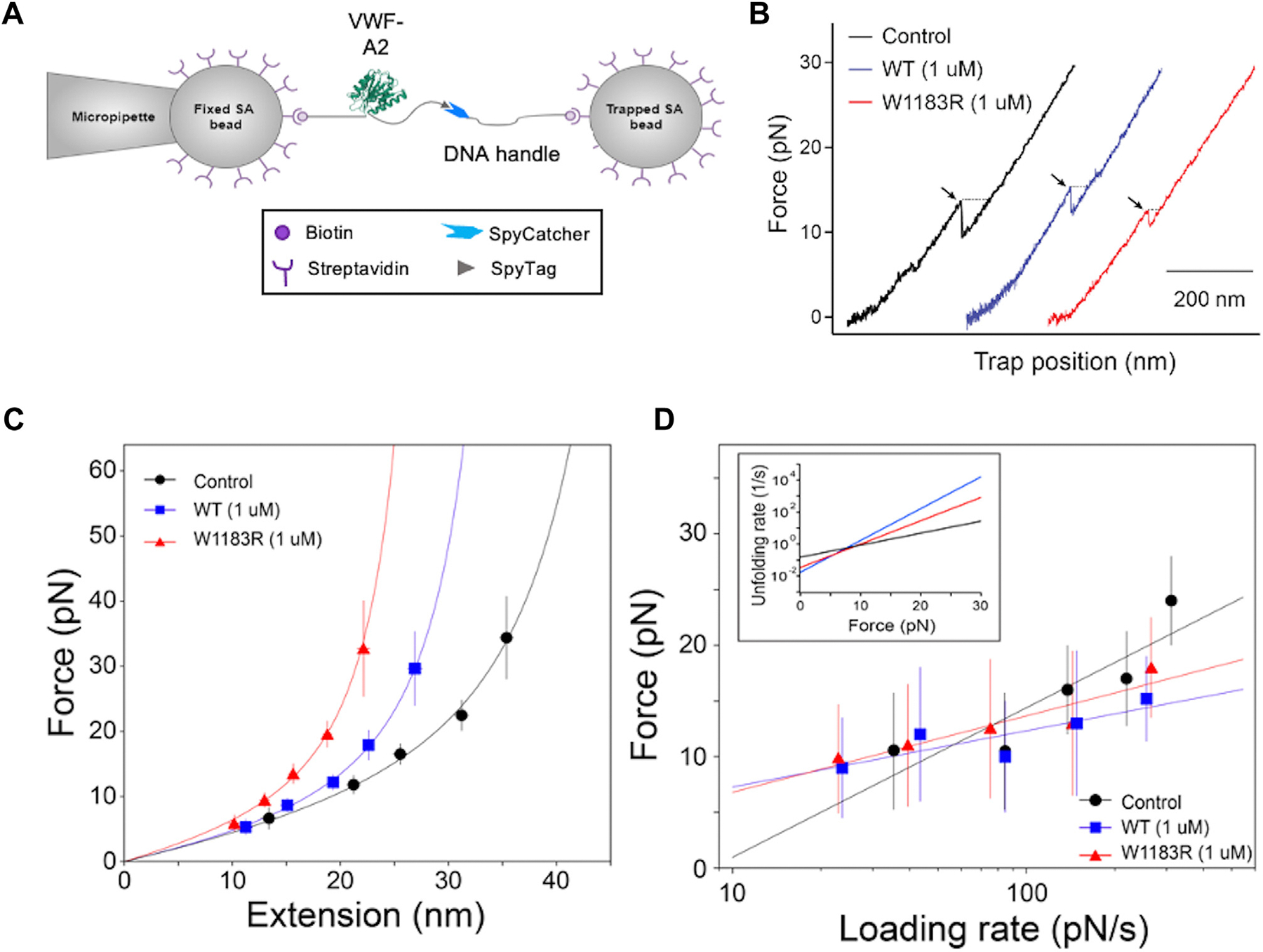
Laser optical tweezer detects conformational changes of von Willebrand factor (VWF) A2 in the presence of a physiological concentration of complement factor H (CFH) proteins. (A) Experimental setup for the laser optical tweezer assay pulling VWF A2 domain tagged with N-terminal biotin and C-terminal SpyTag. (B) Representative force-extension curves generated by pulling at 100 nm/s of the A2 in the absence (a black curve) or presence of 1.0 μM of wild-type CFH (a blue curve) or mutant CFH (a red curve). The arrow in the middle of the curve indicates the evidence of force-induced conformational changes (a sudden drop in the pulling force). Dashed lines with arrows pointed indicate the end-to-end extension associated with protein unfolding. (C) Force-extension profile of the central A2 domain in the absence (a buffer alone) and in the presence of 1.0 μM of wild-type CFH (a blue curve) or mutant (W1183R) CFH (a red curve). The force data shown are the means and the error bars are standard deviation, while the extension data are presented as the mean of the Gaussian fit ± half width at half max height. (D) Plots of unfolding force vs loading rate fit into the Bell-Evans model. Unfolding force data are presented as the center of the tallest bin of the histogram at various loading rates ± the half of the bin width. CFH, complement factor H; VWF, von Willebrand factor.

To gain insight into the molecular mechanism underlying the inhibiting or enhancing effect of CFH proteins, we employed the optical tweezer technology. The experimental setup is illustrated in Figure 4A. As shown, multiple approach-retract force-distance curves were generated as the A2 protein was stretched via the trapped bead. The A2 unfolding is evident by the sudden decrease of force and extension of the trap position, typically occurring between 8 and 20 pN, as indicated by arrows in these curves (Figure 4B). The force and extension of these unfolding events were recorded for further analysis. Following addition of 1.0 μM of WT-CFH or W1183R-CFH protein, the unfolding occurred at similar forces (8–20 pN), but the extension distances were noticeably shorter, as indicated by the dashed lines pointed by the arrows (Figure 4B).

To determine if WT-CFH or W1183R-CFH binding influences the force-induced conformation changes in A2, the most probable unfolding extensions at different unfolding forces were obtained from the pulling experiments under varying speeds or loading rates. The resultant force-extension curves (Figure 4C) were fitted into a WLC model, which yielded the persistence length (Lp) (quantifying the bending stiffness) and contour length (Lc) (describing the maximum extension) for the initially folded structure. The results showed that with an addition of the physiological concentration (~1.0 μM) of WT-CFH or W1183R-CFH, the Lc of the VWF-A2 fragment was significantly altered. Interestingly, the W1183R-CFH resulted in a more significantly shortened Lc in A2 than the WT-CFH did (Table 2). These results demonstrate the significant force-induced conformational changes in VWF A2 following its binding with WT-CFH or W1183R-CFH protein.

To further determine if CFH influences VWF A2 unfolding kinetics, the most probable unfolding forces at various loading rates were identified from the peak of each force distribution histogram and plotted against the logarithm of the loading rates. The unfolding kinetic rates of A2 were extracted using the Bell-Evans model [51,52]. The fitted curves are overlaid as shown in Figure 4D. The best-fit parameters, *k*_*u*_^*0*^ and γ are shown in Table 2. The Bell-Evans model fit revealed an unstressed unfolding rate (*k*_*u*_^*0*^) of 0.15 s^−1^ and an energy barrier position of 0.71 nm for the A2 alone. The energy barrier position parameter γ describes the sensitivity of the protein unfolding rate as a function of force. Therefore, the Bell-Evans model predicts that in the absence of force WT-CFH or W1183R-CFH binding leads to a decrease in the A2 unfolding rate, making part of the A2 structure more stable. However, in the presence of force WT-CFH or W1183R-CFH binding made the A2 more sensitive to force pulling, increasing its unfolding rate more rapidly with force. This prediction is plotted in the inset of Figure 4D. The WT-CFH or W1183R-CFH protein binding made the A2 more stable at forces lower than approximately 8 pN. However, at the forces higher than 8 pN, the A2 becomes less stable (ie, easier to unfold) when either WT-CFH or W1183R-CFH protein is present, resulting in either an inhibitory (by WT-CFH) or an enhancing (by W1183R-CFH) effect on the A2 cleavage by ADAMTS-13 under mechanical force.

Consistent with this hypothesis, at the subphysiological (<120 nM) concentrations of WT-CFH or W1183R-CFH protein, the A2 unfolding with a long (30–50 nm) extension was transformed into 2 unfolding signals with shorter (10–20 nm) extensions (Supplementary Figure S1A). Typically, one unfolding signal occurs at a higher force, like the unfolding force of the single A2 unfolding event in the control (buffer only) case, and another unfolds at a lower force. The WLC equation fits revealed that the Lc of the low- and high-force unfolding events is typically between 25 and 30 nm (Supplementary Figure S1B–E and Table S1). When adding 2 Lc values, the sum matches the value of the single long unfolding of 55.3 nm in the control case (Supplementary Table S1). Therefore, it appears that low subphysiological concentrations of WT or mutant CFH may influence the A2 structure differently in which it transforms its forced unfolding from a one-step process into a two-step process.

### 3.6 | AlphaFold experiments reveal a tight binding interaction between CFH and VWF A2

To gain more insight into how WT-CFH and W1183R-CFH binding may alter the A2 structures, we used AlphaFold2 to simulate the structures of VWF A2 and CFH complexes. CFH exists in both monomeric (majority) and dimeric (up to 15%) forms in solution [54], we simulated the structure of the A2 fragment with both monomeric and dimeric CFH. AlphaFold2 predicts that CFH dimerizes through its SCR 17/18 domains (Supplementary Figure S2A), which is consistent with what was reported in the literature [54,55].

**FIGURE 5:**
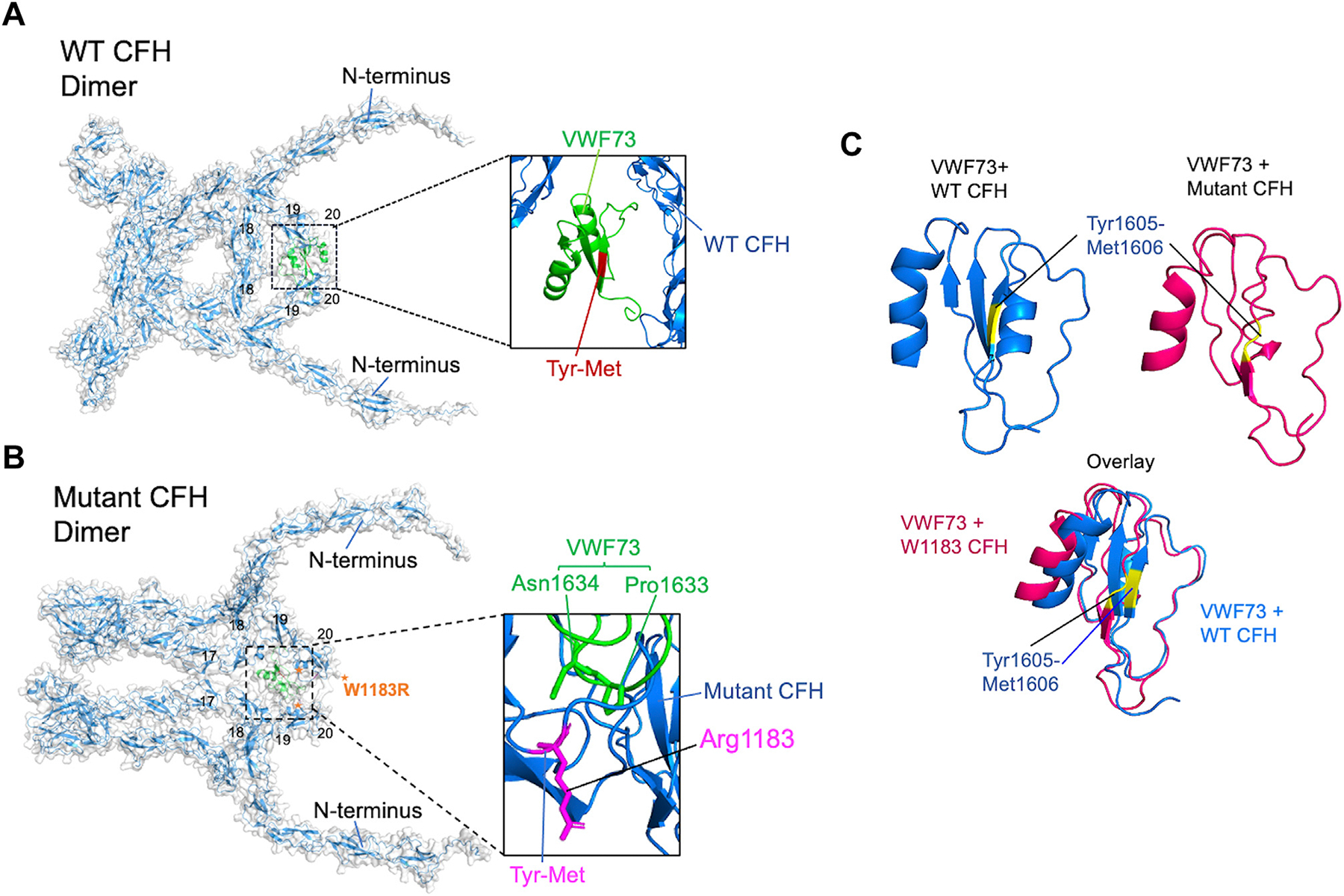
AlphaFold model demonstrates the complexes between VWF73 and dimeric complement factor H (CFH) proteins. (A, B) The AlphaFold models of VWF73 (green) complexed with wild-type and mutant (W1183R) CFH dimer, respectively. ADAMTS-13 cleavage site (Tyr1605-Met1606) is shown in red in the inset of panel A. The cleavage bond and mutation site (Arg1183) are both labeled in magenta in the inset of panel B. (C) An individual VWF73 bound with wild-type CFH or mutant CFH (red), and overlayed structures of both (blue on top of red). The ADAMTS-13 cleavage site (Tyr1605-Met1606) is highlighted in yellow [56]. CFH, complement factor H.

For the VWF73 peptide, AlphaFold2 did not predict a stable interaction between VWF73 peptide and a monomeric CFH (not shown). However, VWF73 forms a stable interaction with a dimeric WT (Figure 5A) [56] or a dimeric mutant CFH (Figure 5B) with high confidence. As shown, the VWF73 has more interactions with a dimeric W1183R-CFH than a dimeric WT-CFH protein. In the mutant, the Arg at 1183 appeared to directly interact with Pro1633 and Asn1634 of VWF at less than 5Å, forming a robust network of hydrogen bonds and hydrophobic interactions (Figure 5B, inset). Another important interface is located near or near the ADAMTS-13 cleavage site, involving in the residues Asn1602-Val1607 and Asp1613-Ile1622 in VWF, and residues Thr1184-Tyr1190 with the mutant CFH protein (not shown). These additional interactions involving in R1183 may alter the conformation of VWF73, leading to significant changes around the ADAMTS-13 cleavage site (Tyr1605 and Met1606) (highlighted in yellow) and converting the originally beta strand (Figure 5C, blue) into a random coil (Figure 5C, red) with an overlayed structure of the 2 VWF73 bound by WT-CFH and W1183R-CFH (Figure 5C, blue and red overlaid). Our AlphaFold2 simulation results provide a possible explanation for why the W1183R-CFH but not WT-CFH may facilitate ADAMTS-13 because the extra Arg at 1183 interacts with VWF-A2, resulting in an altered structure around the ADAMTS-13 cleavage site.

In addition to VWF73, AlphaFold2 also predicted stable interactions between a dimeric CFH protein and the whole A2 domain. The 2 monomers of the dimeric CFH form a large binding pocket through their SCR17-SCR20 domains, which wraps around large binding interfaces on A2, including its α5-helix, β6-strand, and α6-helix structures with one CFH and its α3-helix and α4-less loop with another CFH (Supplementary Figure S2A,B). Without any external forces, it is highly conceivable that the additional binding of CFH would restrict the unfolding of A2 and the exposure of the ADAMTS-13 cleavage site. Therefore, the AlphaFold2 results may potentially explain the protective effect against ADAMTS-13 cleavage on A2.

AlphaFold2 also predicted an interaction between a monomeric WT-CFH and A2, which is relevant to the situation when low concentrations of CFH are present, as shown in Supplementary Figure S1. The monomeric WT-CFH interacts mainly with the portions of A2, including the α3-helix, α5-helix, and β6-strand, potentially leading to a decoupling of this portion from the remaining structure of A2 (Supplementary Figure S2C). The interaction interface between a monomeric W1183R-CFH and a larger C-terminal portion of the A2 domain, including the α4-less loop, β5-strand, α5-helix, β6-strand, and α6-helix (Supplementary Figure S2D). These results could potentially explain the occurrence of a two-step unfolding even of A2 in our laser tweezer assays and the smaller contour lengths for both low and high force unfolding experiments in the presence of a W1183R-CFH under low and subphysiological concentrations as shown in Supplementary Table S1. Together, our structural insights provided by AlphaFold2 elucidate the potential molecular mechanisms underlying the interactions between VWF and CFH. Combined with our in vitro experimental results, these simulations offer new perspectives on how CFH and its mutant protein may affect VWF proteolysis by ADAMTS-13 under mechanical shear forces.

## 4 | DISCUSSION

The present study demonstrates that both WT and mutant CFH proteins interact with various domains of VWF with a high affinity, particularly D’D3, AIM, A1, and A2; the binding of WT-CFH to VWF appears to inhibit VWF proteolysis by ADAMTS-13 under shear, while the binding of W1183R-CFH enhances it. These results are very intriguing because they may offer molecular insight into why plasma VWF multimer size becomes smaller in mice carrying a homozygous mutation that changes Trp (W) to Arg (R) at the amino acid residue of 1206 (*cfh*^*W1206R*^) [57], which corresponds to the amino acid residue of 1183 in human CFH. This may also provide an insight into why VWF–platelet-rich thrombi are not the predominant pathological feature for cHUS resulting from loss-functional mutations in *CFH*. Instead, these patients have fibrin-, but not platelet and VWF-dominant thrombosis in their major organs, resulting in tissue ischemia and damage [58].

How CFH modulates VWF proteolysis by ADAMTS-13 under physiological conditions remains to be poorly understood. Human CFH could be co-purified with VWF [32], suggesting that CFH and VWF association occurs *in vivo*. CFH is primarily produced in hepatocytes [27], but it is also found to be secreted from endothelial cells [2,59,60]. It remains controversial if CFH is stored in the Weibel-Palade bodies of endothelial cells. One study showed that CFH and VWF are colocalized in the Weibel-Palade bodies of cultured endothelial cells [2], but another study showed that CFH and VWF are not colocalized with CFH, which is shown to be present in the cytoplasm, so desmopressin only stimulates the release of VWF but not CFH [60].

In this study, we demonstrate that both WT-CFH and W1183R-CFH proteins bind VWF at various discrete domains including D’D3, AIM-A1 or A1-A2 junction, and A2 with high affinities. Such a direct binding of either WT-CFH or W1183R-CFH to VWF-A2 domain appears to significantly alter the force-induced conformational changes of the central A2 domain. The force-induced conformational changes resulting from W1183R-CFH binding are much greater than from WT-CFH binding, although the exact conformational change in the area containing the sessile bond (Tyr-Met) following binding of WT-CFH vs W1183R-CFH is not fully elucidated.

Similar shear-dependent conformational changes induced by binding of coagulation factor (F)VIII (FVIII) to VWF-A2 were previously reported [36]. In this case, FVIII binding to the A2 domain facilitates the force-induced unraveling of the central A2, allowing ADAMTS-13 to cleave the sessile bond more easily. The mechanism underlying CFH regulation of VWF cleavage may be different from that for FVIII. The binding of both WT-CFH and W1183R-CFH at the no or low pulling forces may overall stabilize part of the structure of A2; at higher forces, CFH protein binding may induce easier A2 unfolding under force. In addition, the W1183R-CFH may interact with the unfolded A2 directly and facilitate the enzymatic activity by altering the conformation of the A2 structure near the cleavage site. Previous studies showed that CFH does not bind to A3 domain directly [2,32]. A hydrogen-deuterium exchange plus mass spectrometric (HX-MS) assay or cryo-EM experiments may be necessary to determine the exact differential conformational changes in or near the central A2 domain upon binding of WT-CFH or W1183R-CFH in our future experiments.

In addition to affecting VWF proteolysis by ADAMTS-13, CFH binding may enhance ristocetin cofactor activity of VWF and ristocetin-induced platelet adhesion and aggregation *in vitro* [2]. CFH and complement factor I (CFI) are 2 negative regulators that are shown to bind ultra-large VWF strings on endothelial surface [59]. This may protect the endothelial surface from assembling and activating complement components including complement factor B, D, and C5, etc [59]. On the other hand, the binding of VWF to CFH enhances CFH activity toward the CFI-mediated degradation of active C3b into inactive iC3b, thus downregulating complement activation [32]. We do not anticipate the mutation in CFH at the complement regulatory module 20 (ie, W1183R) would affect the CFH function in a solution face inactivation of complements, but it clearly affects its negative regulatory activity on the plasma membrane or cell surface. This may result in endothelial damage, inflammation, and thrombosis as seen in patients with cHUS [3,10].

We conclude that our results demonstrate for the first time that the binding of WT-CFH and W1183R-CFH in the terminal complement regulatory module of CFH may differentially affect force-induced conformational changes at or near the ADAMTS-13 cleavage site in the central A2 domain. Such differential conformational changes upon binding of WT-CFH and W1183R-CFH may explain the inhibitory and the enhanced effect, respectively, on VWF proteolysis by ADAMTS-13 under shear. Our findings may shed new light on the pathogenesis of cHUS, which is clearly distinguishable from TTP in terms of the VWF multimer distribution and therapeutic intervention.

## Supplementary Material

MMC1

figs1

fig2

The online version contains supplementary material available at https://doi.org/10.1016/j.jtha.2024.11.031

## Figures and Tables

**TABLE 1:** Kinetic parameters for binding between complement factor H and von Willebrand factor fragments.

VWF Fragments	WT-CFH (*k_D_*)	Mutant (W1183R) (*k_D_*)	Fold^a^
A1-S	No binding	No binding	ND
AIM-A1	3.0 x 10^−9^	3.6 x 10^−9^	0.8
A2	6.1 x 10^−9^	2.3 x 10^−9^	2.7
A1-A2-A3	3.4 x 10^−9^	0.9 x 10^−9^	3.8
D’D3	9.6 x 10^−9^	2.6 x 10^−9^	3.7
D’D3-A1	6.8 x 10^−9^	3.0 x 10^−9^	2.3
D’D3-A1-A2	9.6 x 10^−9^	5.5 x 10^−9^	1.7

A1-S, A1 short; CFH, complement factor H; *k_D_*, dissociation constant; ND, not determined; VWF, von Willebrand factor.

a Fold of affinity change comparing mutant with wild-type.

**TABLE 2:** Contour length and persistent length of VWF-A2 in the absence or in the presence of 1.0 μM of complement factor H.

Parameters	No CFH	WT-CFH	Mutant CFH
Lc (nm)	55,3	40,0	31,8
Lp (nm)	0.28	0.38	0.38
k_U_^0 61^	0.15	0.017	0.034
γ (nm)	0.71	1,88	1,38

K_U_^0^, Unstressed unfolding rate; L_c_, the contour length of the whole structure; L_p_, persistent length; mutant CFH, W1183R complement factor H; WT-CFH, wild-type complement factor H; ץ_u_, barrier position.
