# Supplementary material for: A mutant complement factor H (W1183R) enhances proteolytic cleavage of von Willebrand factor by ADAMTS-13 under shear
Source: J Thromb Haemost. Author manuscript; Available in PMC 2026 Apr 28. (PMC13123425; doi:10.1016/j.jtha.2024.11.031)
Supplement: MMC1 [file NIHMS2161414-supplement-MMC1.pdf]

Table S1. Contour length and persistent length of VWF-A2 in the absence or in the presence of various concentrations of complement factor H (CFH) proteins

Parameters	Buffer	12 nM (low force)		12 nM (high force)		120 nM (low force)		120 nM (high force)	
		WT	W1183R	WT	W1183R	WT	W1183R	WT	W1183R
L_c (nm)	55.3	28.93	26.01	29.98	24.25	27.20	26.33	33.56	35.49
L_p (nm)	0.28	0.76	0.70	0.55	0.89	1.09	1.18	0.41.	0.50
K_u^0 (S^{-1})	0.15	0.11	0.12	0.11	0.034	0.0001	0.14	0.14	0.014
Y_u(nm)	0.71.	1.77	1.60	0.99.	1.16	6.23	2.17	0.48	2.20

WT, wild type factor H; W1183R, mutant factor H; L_c, the contour length of the whole structure; L_p, persistent length. K_U^0: Unstressed unfolding rate; γ_u: barrier position
