# Supplementary figures and images for: A mutant complement factor H (W1183R) enhances proteolytic cleavage of von Willebrand factor by ADAMTS-13 under shear
Source: J Thromb Haemost. Author manuscript; Available in PMC 2026 Apr 28. (PMC13123425; doi:10.1016/j.jtha.2024.11.031)

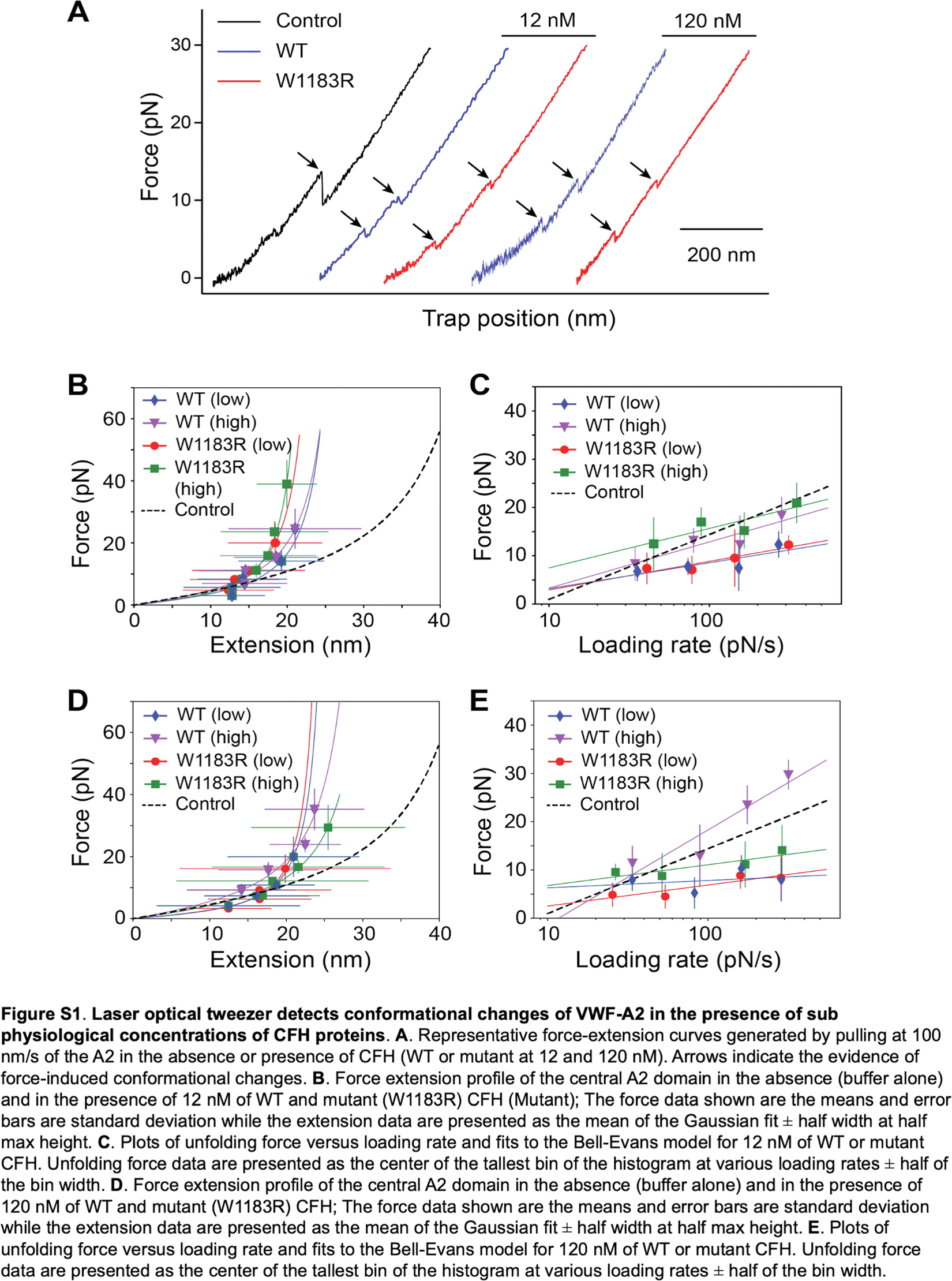

Supplement: figs1 [file NIHMS2161414-supplement-figs1.jpg]

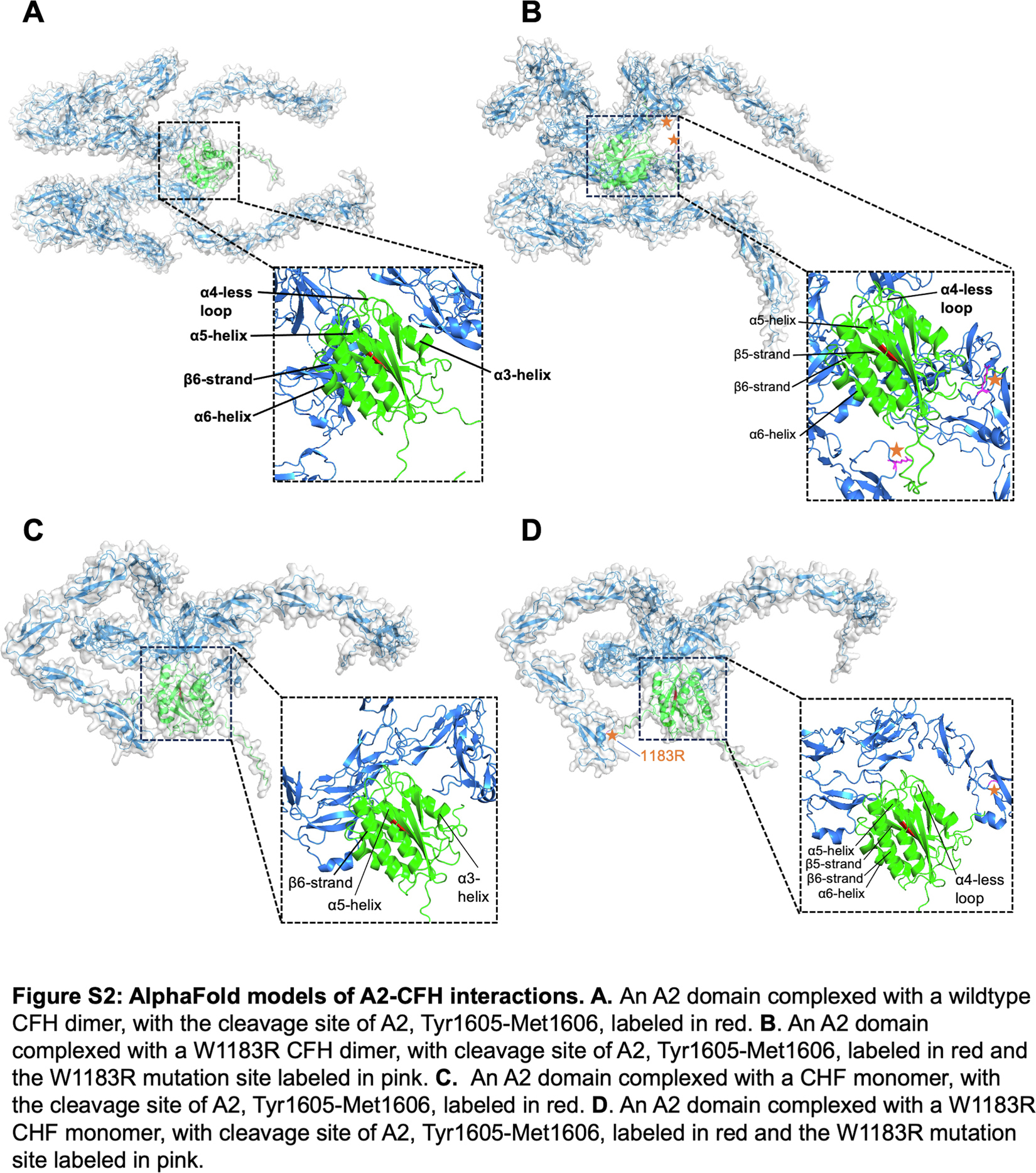

Supplement: fig2 [file NIHMS2161414-supplement-fig2.jpg]
